# Observations on Obstetrics in General Practice

**Published:** 1904-04

**Authors:** 


### OBSERVATIONS ON OBSTETRICS IN GENERAL PRACTICE.*

The art of assisting women in childbirth differs but little in general country practice from city private practice. The general

*Read by J. F. McNulty, M.D., Callaway, Neb., before the Custer County Medical Society, Broken Bow, Neb., February 9, 1904.

practitioner is working at a disadvantage, as he is frequently called without a moment's notice, while the specialist has generally a line on his case during the whole period of gestation. The general practitioner is very frequently brought face to face with serious and alarming situations without notice, and quite often without sufficient preparation. The ability to successfully cope with what may be called emergency cases requires a cool head and rapid judgment. He must have the faculty of doing nothing at the wrong time, and the ability to act promptly at the right time. We should constantly keep in mind the fact that meddlesome midwifery is poor midwifery. We are called simply because it may become necessary to assist nature in the expulsion of the products of conception, but with the distinct understanding and hope that our efforts will not be required.

1. We may become meddlesome by rupturing the membranes too soon. Show me a man who has a record for using the forceps, who, in fact, is forced to use them often, and I will show you a man who is a chronic premature rupturer of the amniotic sac.

2. We can, and unfortunately do, become meddlesome, by introducing diseased germs into a very fine culture medium—the contents of the human uterus. By cleanliness and the strict attention to the details of antiseptic surgery much trouble may be avoided. I am now attending a case of puerperal fever where the attending physician neglected to prescribe even a carbolic acid wash. The attending physician should prepare as for a surgical operation in every instance. There should be no situation which would call for a hasty examination without first giving marked attention to the preparation of your hands. The obstetrical practitioner of to-day should always remember that he is, for the time being, at least, a surgeon when called upon to use any kind of instrument.

3. We can, and do, become meddlesome by neglect—neglect to repair a laceration immediately. The gynecologists feast upon the neglect of the obstetrical practitioner. Nine women out of ten who consult us for private trouble date their period of suffering from a particular confinement or a particular miscarriage. You have in a laceration a fresh wound. Sew it up; dress it surgically, and no trouble follows. If not sufficiently large to require a ligature, merely apply an antiseptic dressing instead of allowing it to ulcerate and become an avenue of infection.

Few persons die during an operation; few women die during delivery; but the effects resulting from operations and deliveries give us deaths by the thousands. Suppose the case of puerperal fever of which I spoke should die, or should recover and later develop pus tubes, and the removal of them cause death, where should the primary cause of death be placed?

Neglected abrasions of the vaginal mucous membrane, slight lacerations of the cervix with more or less tearing of the pelvic floor, is, I believe, the primary cause of the major portion of female complaints. We should see that the various organs after delivery are placed in the position which nature intended them to occupy. More tissue should be placed to support the uterus than just sufficient to afford covering for the contents of the rectum. We must answer for a great many prolapses, not owing to what we have done, but what we have left undone.

My old professor, Dr. Earle, of the College of Physicians and Surgeons, Chicago, was in the habit of saying: "Remember, boys, you can not prevent a child from being born." The child will be born, as a rule, in spite of us, but it is very essential that we have at our finger tips the normal diameters of the pelvis and the fetal head; also the positions the fetal head may assume in passing through the parturient canal. These points are called the alphabet of obstetrics, and every physician should have a mental picture of the situation, no matter what position the child's head may be forced to assume. The ability to properly and accurately follow the fetal head comes by painstaking observation. How few of us endeavor to make out the various positions and mentally review the diameters while attending a case. I think it good practice to go over this old alphabet in every case, no matter how normal it may be. When we come then in contact with an abnormal situation, we at once recognize it, and can prepare to meet it promptly. If we find that natural efforts are not sufficient to meet the new situation, and artificial aid is absolutely called for, then act without delay—giving ample time for thorough preparation, however.

Offer no excuse for using forceps. When indicated use them just as you would a certain drug if indicated in a given case. I was recently obliged to use forceps in a case where the membranes had ruptured two days previous to the beginning of actual labor pains. The mother was a large, healthy and strong woman, twenty-eight years of age, this being her second child. The occiput was found to be in the left posterior position. It failed to make the turn, and remained fixed. The patient became exhausted, and the natural efforts were such that it was evident artificial aid would absolutely be required. After thorough preparation the patient was chloroformed, forceps were placed in position, and the child delivered. By removing the forceps before the fetal head has passed over the perineum we avoid the lacerations which sometimes occur when delivery takes place with the forceps in position. I believe it is not good practice to endeavor to rotate the head of fetus with forceps in position, although we are at times advised to do so. Upon making the usual call the next day I found patient to have a degree of fever. I frequently find a slight fever on second or third day, to which I pay no attention. There is a decided something between the fever of sepsis and that of exhaustion or reaction. We may do harm by ordering vaginal injections in such cases. However, when the discharge is offensive, with fever or without, I invariably use vaginal douches. If no rapid and decided improvement takes place, irrigate the uterus, and in every case inspect cervix and vagina.

There is no class of cases where symptoms vary more widely than in premature deliveries, although the cause is the same in each. If, in such cases, I find the os dilated, pain and hemorrhage, the prospect of saving the child is poor, indeed. Driving, as we do, many miles in the country to see such patients, I have made it a practice to rapidly dilate if necessary, and turn out the entire contents of the uterus. The prospect of hemorrhage following a complete operation of this character is slight. The danger in those cases comes from leaving some of the after-birth intact.

Here, again, the trust-to-luck obstetrician is apt to have a sudden or remote death on his conscience.

It is very essential that we manage breech presentations with great caution. Until the breech appears in the vulva there is, as a rule, no danger to the child. But we must act quickly when the breech appears. If we have hastened labor by force, there is the danger of the head approaching the brim of the pelvis before dilatation is fully established. If dilatation is not fully established, then the labor will be most difficult. Force and haste on our part, when neither is called for, is the great difficulty in those cases. The temptation is great. Avoid it. The lower uterine segment offers the obstruction in a large majority of those cases when we are inclined to attribute the cause to a contracted pelvis. Deliver both upper extremities with the body of child. Place forearm under the body, and fingers in child's mouth and make traction on breech. Force should be exerted toward the pelvis of child to avoid fracture of the femur. I have never been obliged to place forceps on breech to aid delivery.

Let us shortly consider a few diseases that influence the course of pregnancy.

Abortion and premature labor may be caused by an attack of pneumonia. Pneumonia, during the last few months of pregnancy, is especially to be dreaded, as fully one-half the cases die. Hence our prognosis should be grave. If the mother, in such a case, is threatened with asphyxia, we are justified in causing premature delivery.

Pleurisy may cause premature delivery, but the course is not influenced by pregnancy to any great extent.

The tubercle of consumption will, if the general condition of the patient is good, remain in a latent state during pregnancy, but when the patient is debilitated the disease generally breaks out with renewed vigor. Such persons should not be permitted to nurse the child.

Autointoxication arises from sluggish action of the kidneys and liver. The earlier in pregnancy the lesion of the kidneys manifests itself, the more serious for both mother and child.

I am in the habit of sitting by the bedside in every instance and occasionally kneading the uterus—to insure contraction—for one full hour after the delivery of the placenta.

Never leave the house while the woman has a pulse of over 100. When we feel hard, firm contraction, with a steady pulse and a temperature below 100°, we may turn to our couch and sleep the sleep of sometimes profound exhaustion.—*Western Medical Review.*

